# Expression of Concern: Contribution of labor related gene subtype classification on heterogeneity of polycystic ovary syndrome

**DOI:** 10.1371/journal.pone.0339143

**Published:** 2025-12-18

**Authors:** The PLOS One Editors

The *PLOS One* Editors issue this Expression of Concern because this article [1] was identified as one of a series of submissions for which we have concerns about the peer review process. Readers are advised to interpret the article [1] with caution.

The Editors have no evidence of any author involvement in the peer review concerns.

Additionally, the article cited as Reference 58 was retracted after [1] was published, and the article cited as Reference 34 was retracted before [1] was published.
