# Supplementary figures and images for: Contribution of labor related gene subtype classification on heterogeneity of polycystic ovary syndrome
Source: PLoS One. 2023 Mar 1;18(3):e0282292. doi: 10.1371/journal.pone.0282292 (PMC9977056; doi:10.1371/journal.pone.0282292)

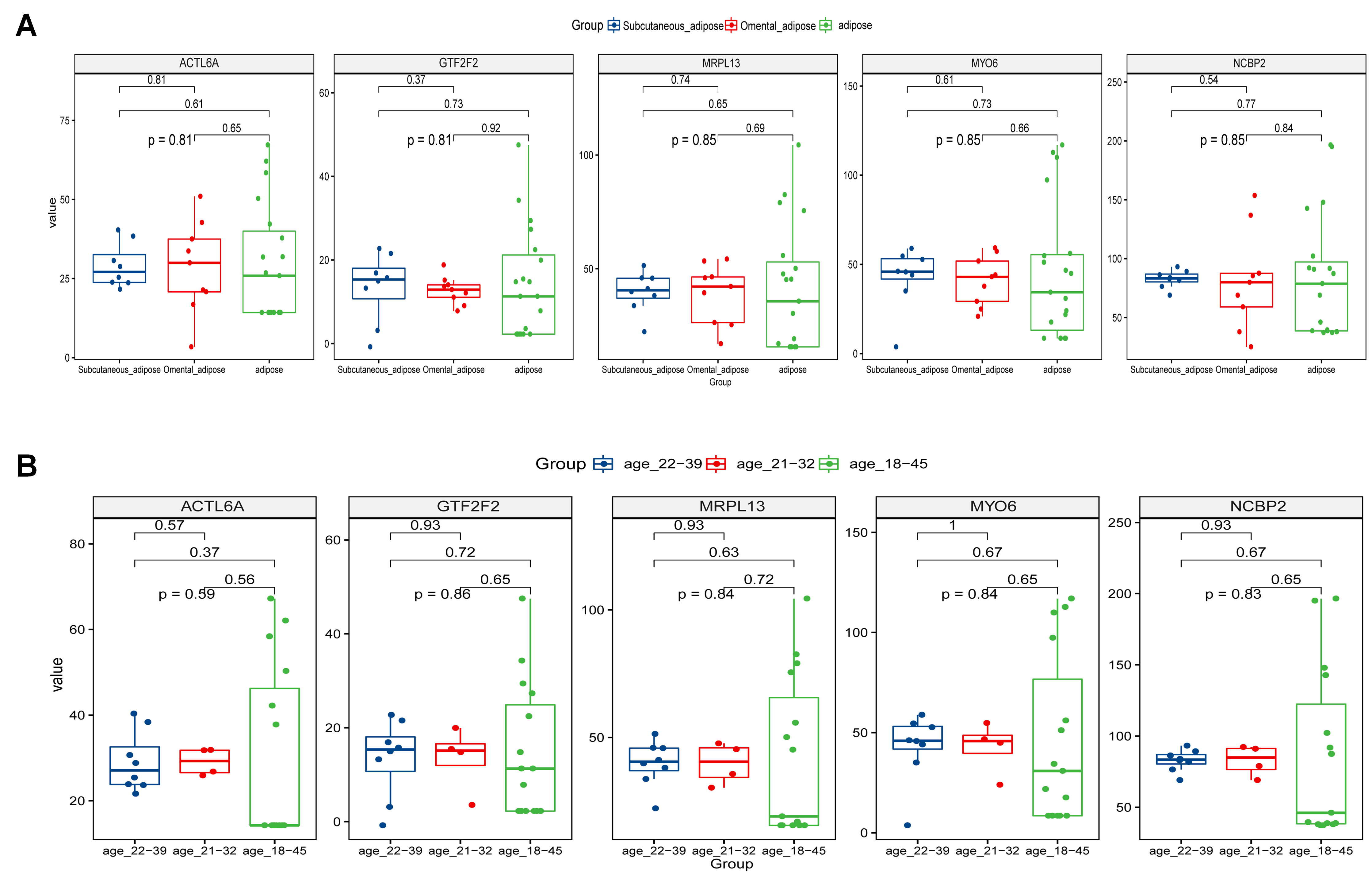

Supplement: S1 Fig — The expression of these hub genes was shown among adipose tissue subtypes (A) and various age subgroups (B). (TIF) [file pone.0282292.s001.tif]
