# Supplementary material for: Contribution of labor related gene subtype classification on heterogeneity of polycystic ovary syndrome
Source: PLoS One. 2023 Mar 1;18(3):e0282292. doi: 10.1371/journal.pone.0282292 (PMC9977056; doi:10.1371/journal.pone.0282292)
Supplement: Attachment — Submitted filename: Response to Reviewers.doc [file pone.0282292.s002.doc]

**RE: PLOS ONE - Decision on No.PONE-D-22-28834**

**Title: Contribution of labor related gene subtype classification on heterogeneity of polycystic ovary syndrome**

Dear Dr. Laganà:

Thank you for inviting a revision of our manuscript, which we are now submitting for consideration for publication.

We would like to thank you for the constructive and positive comments. We have revised the manuscript in response to the suggestions of reviewers where appropriate. The corrections and amendments have been highlighted RED in the revised manuscript. We also provided point-by-point replies to each concern raised by the reviewers and editor as listed below. The revised manuscript has been uploaded in the submission system of the journal.

We believe that our revised manuscript is greatly improved, and we hope that it is now more acceptable for publication in *PLOS ONE.*

Thank you.

With best wishes!

Sincerely,

Fang-Fang Wang

**Replies to the comments**

Please include the following items when submitting your revised manuscript:

1. A rebuttal letter that responds to each point raised by the academic editor and reviewer(s). You should upload this letter as a separate file labeled 'Response to Reviewers'.
2. A marked-up copy of your manuscript that highlights changes made to the original version. You should upload this as a separate file labeled 'Revised Manuscript with Track Changes'.
3. An unmarked version of your revised paper without tracked changes. You should upload this as a separate file labeled 'Manuscript'.

**Journal Requirements:**

When submitting your revision, we need you to address these additional requirements.

1. Please ensure that your manuscript meets PLOS ONE's style requirements, including those for file naming. The PLOS ONE style templates can be found at

https://journals.plos.org/plosone/s/file?id=wjVg/PLOSOne_formatting_sample_main_body.pdf and

https://journals.plos.org/plosone/s/file?id=ba62/PLOSOne_formatting_sample_title_authors_affiliations.pdf

**Response:** Thank you for the comment! As suggested, we have ensured that the revised manuscript meets PLOS ONE's style requirements.

2. We note that the grant information you provided in the ‘Funding Information’ and ‘Financial Disclosure’ sections do not match.

When you resubmit, please ensure that you provide the correct grant numbers for the awards you received for your study in the ‘Funding Information’ section.

**Response:** Thank you for the comment! As suggested, we have ensured that we have provided the correct grant numbers in the “Funding Information” section in the submission system.

This study was supported by the National Natural Science Foundation of China (81873837 to Fangfang Wang and 81973900 to Jue Zhou). The funders had no role in study design, data collection and analysis, decision to publish, or preparation of the manuscript. The funding information was provided in the file “Funding statement” which has also been uploaded in the submission system.

3. In your Data Availability statement, you have not specified where the minimal data set underlying the results described in your manuscript can be found. PLOS defines a study's minimal data set as the underlying data used to reach the conclusions drawn in the manuscript and any additional data required to replicate the reported study findings in their entirety. All PLOS journals require that the minimal data set be made fully available. For more information about our data policy, please see http://journals.plos.org/plosone/s/data-availability.

Upon re-submitting your revised manuscript, please upload your study’s minimal underlying data set as either Supporting Information files or to a stable, public repository and include the relevant URLs, DOIs, or accession numbers within your revised cover letter. For a list of acceptable repositories, please see http://journals.plos.org/plosone/s/data-availability#loc-recommended-repositories. Any potentially identifying patient information must be fully anonymized.

Important: If there are ethical or legal restrictions to sharing your data publicly, please explain these restrictions in detail. Please see our guidelines for more information on what we consider unacceptable restrictions to publicly sharing data: http://journals.plos.org/plosone/s/data-availability#loc-unacceptable-data-access-restrictions. Note that it is not acceptable for the authors to be the sole named individuals responsible for ensuring data access.

We will update your Data Availability statement to reflect the information you provide in your cover letter.

Response: Thank you for the comment! As suggested, we have added the URLs of the resource of RNA-seq and microarray in the part of data availability statement of the revised manuscript.

Additional Editor Comments:

The topic of the manuscript is interesting. Nevertheless, the reviewers raised several concerns: considering this point, I invite authors to perform the required major revisions.

[Note: HTML markup is below. Please do not edit.]

Reviewers' comments:

Reviewer's Responses to Questions

Comments to the Author

1. Is the manuscript technically sound, and do the data support the conclusions?

The manuscript must describe a technically sound piece of scientific research with data that supports the conclusions. Experiments must have been conducted rigorously, with appropriate controls, replication, and sample sizes. The conclusions must be drawn appropriately based on the data presented.

Reviewer #1: Yes

Reviewer #2: Yes

2. Has the statistical analysis been performed appropriately and rigorously?

Reviewer #1: No

Reviewer #2: Yes

3. Have the authors made all data underlying the findings in their manuscript fully available?

The PLOS Data policy requires authors to make all data underlying the findings described in their manuscript fully available without restriction, with rare exception (please refer to the Data Availability Statement in the manuscript PDF file). The data should be provided as part of the manuscript or its supporting information, or deposited to a public repository. For example, in addition to summary statistics, the data points behind means, medians and variance measures should be available. If there are restrictions on publicly sharing data—e.g. participant privacy or use of data from a third party—those must be specified.

Reviewer #1: Yes

Reviewer #2: Yes

4. Is the manuscript presented in an intelligible fashion and written in standard English?

PLOS ONE does not copyedit accepted manuscripts, so the language in submitted articles must be clear, correct, and unambiguous. Any typographical or grammatical errors should be corrected at revision, so please note any specific errors here.

Reviewer #1: No

Reviewer #2: Yes

5. Review Comments to the Author

Please use the space provided to explain your answers to the questions above. You may also include additional comments for the author, including concerns about dual publication, research ethics, or publication ethics. (Please upload your review as an attachment if it exceeds 20,000 characters)

Reviewer #1: I read with great interest the Manuscript titled “Contribution of labor related gene subtype classification on heterogeneity of polycystic ovary syndrome” (PONE-D-22-28834), which falls within the aim of this Journal.

In my honest opinion, the topic is interesting enough to attract the readers’ attention. Methodology is accurate and conclusions are supported by the data analysis.

Nevertheless, authors should clarify some point and improve the discussion citing relevant and novel key articles about the topic.

Authors should consider the following recommendations:

1. Manuscript should be further revised by a native English speaker.

**Response:** Thank you for the comment! As suggested, we have invited a native English speaker to improve the language of the present manuscript.

2 - In the Results section, the Authors have simply reported the p values, from which however it is not possible to deduce the real clinical relevance of the highlighted statistical significance. In order to better understand the obtained results, I suggest reporting not only the p values, but also the corresponding confidence intervals.

**Response:** Thank you for the comment! As suggested, we have mentioned the p-value was set to less than 0.05, and the corresponding confidence intervals were 95% in the revised manuscript.

3- Does this manuscript conform the Enhancing the QUAlity and Transparency Of health Research (EQUATOR) network guidelines? It would be mandatory to declare about this element.

**Response:** Thank you for the comment! As there is no available study type in the reporting guidelines for the present study, we did not declare about this element in the original version, however, we have fully considered these guidelines when reporting the present study.

4- I recommend to highlight, at least briefly, current management strategies for PCOS (PMID: 35472446; PMID: 3407070) and reproductive/obstetric outcomes in this population (PMID: 33876903).

**Response:** Thank you for the comment! Yes, we fully agree with this. As suggested, we have highlighted the current management strategies for PCOS and the reproductive/obstetric outcomes in this population in the revised manuscript. We also added the three papers (PMID: 35472446; PMID: 3407070; PMID: 33876903) as new references in the revised manuscript.

Reviewer #2: The manuscript is well written and complete.

My suggestions are:

1. - How your results can affect the clinical practice? Is a genetic test feasible and useful in these patients? Is it economically sustainable?

**Response:** Thank you for the comment! As suggested, we have added the contents in the discussion part of the revised manuscript.

2.- If a patient have a genetic predisposition, what is the strategy to reduce risk of preterm birth?

**Response:** Thank you for the comment! As suggested, we have added the contents in the discussion part of the revised manuscript.

3. Results are very interesting but very limited if they don't improve outcome. Please, you have to explain the primary and secondary prevention methods that can be applied in these patients.

**Response:** Thank you for the comment! As suggested, we have added the contents in the discussion part of the revised manuscript.

MINOR REVISIONS

- Page 5, lines 96,97: please correct “preterm” labour and add space between “genes” and “was”

- Page 8, line 158: please correct “genes” not gene

- Page 12, line 250: please correct “genes” not gens

**Response:** Thank you for the comment! As suggested, we have revised all of the contents above in the revised manuscript.

6. PLOS authors have the option to publish the peer review history of their article (what does this mean?). If published, this will include your full peer review and any attached files.

If you choose “no”, your identity will remain anonymous but your review may still be made public.

Do you want your identity to be public for this peer review? For information about this choice, including consent withdrawal, please see our Privacy Policy.

Reviewer #1: Yes: Alessandra Lopez

Reviewer #2: Yes: Basilio Pecorino

[NOTE: If reviewer comments were submitted as an attachment file, they will be attached to this email and accessible via the submission site. Please log into your account, locate the manuscript record, and check for the action link "View Attachments". If this link does not appear, there are no attachment files.]

While revising your submission, please upload your figure files to the Preflight Analysis and Conversion Engine (PACE) digital diagnostic tool, https://pacev2.apexcovantage.com/. PACE helps ensure that figures meet PLOS requirements. To use PACE, you must first register as a user. Registration is free. Then, login and navigate to the UPLOAD tab, where you will find detailed instructions on how to use the tool. If you encounter any issues or have any questions when using PACE, please email PLOS at figures@plos.org. Please note that Supporting Information files do not need this step.
